# Glabridin triggers over-expression of apoptosis inducing factor (AIF) gene in *Candida albicans*

**DOI:** 10.18502/cmm.4.3.172

**Published:** 2018-09

**Authors:** Maryam Moazeni, Mohammad Taghi Hedayati, Mojtaba Nabili

**Affiliations:** 1 Invasive Fungi Research Center, Mazandaran University of Medical Sciences, Sari, Iran; 2 Department of Medical Mycology, School of Medicine, Mazandaran University of Medical Sciences, Sari, Iran; 3 Faculty of Medicine, Sari Branch, Islamic Azad University, Sari, Iran

**Keywords:** AIF gene, Apoptosis, Candida albicans, Glabridin

## Abstract

**Background and Purpose:**

*Candida albicans* is a prevalent human fungal pathogen that can cause a wide spectrum of diseases, from superficial mucosal infections to systemic disorders, in patients with impaired immunity. Glabridin is a pyranoisoflavan originally extracted from root extract of *Glycyrrhiza glabra*. Glabridin can also mediate apoptosis in yeast cells by changing the mitochondrial membrane potential, activation of caspase-like proteases, and DNA cleavage. The aim of this study was to investigate the mechanism of action of glabridin in *C. albicans*.

**Materials and Methods:**

*Candida albicans* ATCC14053 was applied as the standard strain. Total RNA was extracted from the isolate under glabridin-treated and untreated conditions. To evaluate the alternations in the apoptosis inducing factor (*AIF*) gene expression, real-time polymerase chain reaction (real-time -PCR) was performed, and the obtained data were analyzed using REST software.

**Results:**

Expression of the *AIF* gene was represented as the ratio of expression relative to the reference gene. According to the REST^®^ output, the expression of the *AIF* gene increased significantly (*P<0.05*) under the glabridin-treated condition.

**Conclusion:**

Our results suggested that glabridin may induce apoptosis through the caspase-independent route and might be considered as an anti-*Candida* agent.

## Introduction

*Candida albicans* is a prevalent human fungal pathogen that can cause a wide spectrum of diseases, from superficial mucosal infections to systemic disorders, in patients with impaired immunity [1]. The mortality and morbidity rates are reported from 40 to 80%, especially in the bone marrow and organ transplant recipients [2]. Although *C. albicans* is mostly the causative agent of candidiasis, other species including *C. glabrata*, *C. parapsilosis*, *C. krusei*, and *C. tropicalis* are also involved in this condition [3]. The emergence of drug-resistant strains has become a major medical concern due to the limitations in clinical availability of the antifungal drugs [4]. Accordingly, the discovery of novel antifungal agents/ formulations seemed to be necessary [5, 6]. 

Nowadays, natural products have gained increasing attention for potential use against drug-resistant fungi [7]. Glabridin (Gla) is a pyranoisoflavan originally isolated from root extract of *Glycyrrhiza* *glabra* plant (licorice).

Previous studies have shown that Gla has different pharmacological activities such as antimicrobial activity, cytotoxic activity, anti-inflammation, anti-proliferative, anti-obesity effect, and prevention of osteoporosis [8-11]. Apoptosis is the process of programmed cell death (PCD) that is essential for the protection of health and tissue function in organisms. Gla can also mediate apoptosis in yeast cells by changing in mitochondrial membrane potential, activation of caspase like proteases, and DNA cleavage [12, 13]. In yeasts, apoptosis is elucidated by two distinct routes, namely caspase-dependent and caspase-independent manners. According to the evidence, apoptosis-inducing factor (*AIF*), AIF-homologous mitochondrion-associated inducer of death, and endonuclease G (EndoG) can all induce apoptotic cell death in a caspase-independent manner [14]. 

Our research focused on designing a new agent using natural products. The aim of this study was to investigate the mechanism of action of the glabridin in *C. albicans* by evaluating the alternation in the expression of the *AIF* gene.

## Materials and Methods

***Fungal strain and cultures***

*Candida albicans* ATCC14053 was grown on the Sabouraud Dextrose Agar (SDA) medium (Difco, USA) with chloramphenicol and incubated at 30°C for 24 h. The strain was previously identified by DNA sequencing of the complete ribosomal DNA internal transcribed spacer (rDNA-ITS) region.

**Table 1:** Primers used for *AIF* and *ACT1* gene in PCR

**Gene**	**Accession no./identifier**	**Primer Name**	**Primer Sequence**	**PCR product length (bp)**
*AIF*	orf19.3362	AIF-F AIF-R	CAATGCCGACACCATAAGTG TCGTTCGAGTGAGTCCTGTG	183
*ACT*	FN435840.1	ACT1-F ACT1-R	TTCCAGCCTTCTACGTTTCC GAATCGACCTTGCTGGTAGA	164

***Extraction and measurement of RNA***

Total RNA was extracted using an RNAX-Plus Kit (SinaClon, Karaj, Iran) from a standard strain of *C. albicans* under Gla-treated and untreated conditions according to the manufacturer’s instructions. In order to achieve a large mass of *C. albicans* cells, the yeasts were treated with the sub-minimum inhibitory concentration (MIC) concentration of Gla followed by AFST performed in 24-well macrodilution trays. A positive control (untreated *C. albicans*) was also run for each isolate in each plate. The concentrations and purity of RNA samples were obtained by spectrophotometric measurements using a Biochrom WPA Bio-wave II spectrophotometer (Biochrom Ltd., Cambridge, UK). To remove contamination with gDNA, equal concentrations of RNA treated with DNase were subjected to cDNA synthesis using the PrimeScriptTM RT Reagent Kit (Vivantis, Shah Alam, Malaysia) according to the manufacturer’s instructions.

***Primer designing and characterization of the AIF gene***

Primers were designed on the basis of the annotated sequence of *AIF* gene obtained from *Candida* Genome Database (CGD) (Table 1). As the sequence of *AIF* was not characterized and not published in the NCBI database, the sequence of the gene was registered in the NCBI database under accession number MH998527.

***Evaluating the alternation of AIF gene expression***

The beta-actin gene (*ACT1*) was used as an endogenous reference gene. Standard curves for each gene were established with four serially diluted cDNA, which was obtained from cells grown to mid-log phase at 37°C by using specific primers and under appropriate polymerase chain reaction (PCR) conditions. Real-time PCR was performed with a StepOne ABI Real-Time PCR System (ABI, US), and SYBR Premix Ex Taq II (Takara, Shiga, Japan) was used as a reagent specifically designed for intercalator-based real-time PCR. 

All the PCR mixtures contained the following: 10 mL of SYBR Premix Ex Taq II (2×), 2 mL of first-strand cDNA, 0.4 mM of each primer, and dH_2_O to a final volume of 20 mL. The PCR amplification program consisted of an initial denaturation step at 95°C for 30 s, followed by 40 cycles each consisting of two steps of 95°C for 5 s and 60°C for 30 s. Negative controls were included in each run. Expression of all the genes was normalized to the housekeeping gene *ACT1* and was analyzed using REST^®^ (2009) software (QIAGEN; http://www.gene-quantification.com/rest-2009.html), which uses the comparative Ct method (ΔΔCt) to analyse data. Experiments under each condition were performed in duplicate on two different days to assess reproducibility.

***Statistical analysis***

Statistical analysis was performed by using the REST^®^ software. The software uses pair-wise fixed reallocation randomisation test, and *P-values *less than 0.05 were considered statistically significant. Results are presented as means and standard deviations. 

## Results and Discussion

***Quantitative Real-time RT-PCR assay***

**Figure 1:**
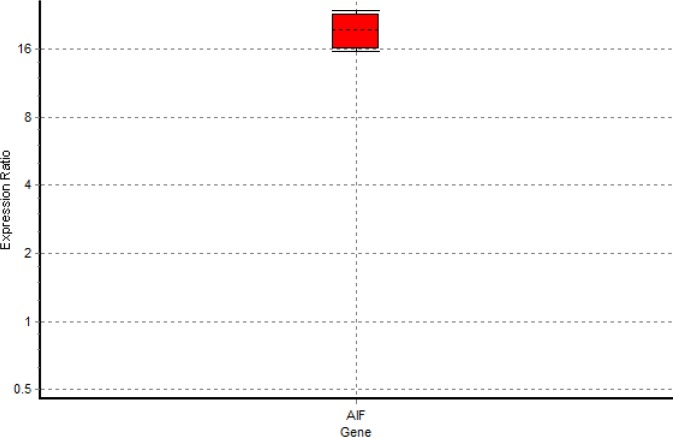
Expression ratio of AIF apoptotic genes. Relative gene expression is the ratio of expression under Gla-treated condition relative to that of the untreated condition. Values between 0 and 1 indicate underexpression, while values >1 indicate overexpression when the data are normalized to the selected housekeeping gene.

The A260 /A280 ratio of the extracted RNA was read between 1.8 and 2 by a spectrophotometer, which indicates the acceptable purity of the RNA. *ACT1* was applied as an internal control gene or an endogenous reference gene. *AIF* primers had similar efficiency in a titration experiment using *C. albicans* cDNA (1000 ng – 1000 pg) in serial dilutions (data not shown). Expression of *AIF* gene was represented as the ratio of expression relative to that of untreated log-phase yeast. The REST software was used to show the relative expression between treated and untreated (control) samples of the studied gene. The boxplot obtained from REST 2009 provides good information for clarifying gene expression data. It consists of the smallest observation (sample minimum), lower quartile, median, upper quartile, and largest observation (sample maximum). Values between 0 and 1 demonstrate low expression, while values >1 indicate high expression. According to the REST^®^ output, expression of the *AIF* gene increased remarkably (*P<0.05*; Table 2). As Figure 1 depicts, the ratio of gene expression under Gla-treated condition is significantly higher than that of untreated condition. 

Recently, antifungal resistance has been rising despite the progress achieved in this regard. This has led researchers to attempt new sources of drugs, such as plant sources [15]. Medicinal plants are widely used in traditional medicine in various medical fields. *Glycyrrhiza glabra* is a well-known herbal medicine found throughout the world. It is one of the oldest herbs widely used in traditional medicine and was also used as a flavor to conceal the unpleasant odor of other drugs [16]. There are few studies regarding the inhibitory effects of Gla, the main and most effective substrate of the extract which is also synthesized commercially, on *C. albicans.* These studies are limited to the effects of this compound on the growth of *C. albicans* [8].

**Table 2:** Expression pattern of *AIF* gene in *C. albicans*

**Gene**	**Type**	**Reaction Efficiency**	**Expression**	**Std. Error**	**95% C.I.**	**P(H1)** ^*^	**Result**
*AIF*	TRG	1.0	19.226	15.824 - 23.366	15.604 - 23.692	0.000	UP
*ACT*	REF	1.0	1.000				

* P(H1) - Probability of alternate hypothesis that difference between sample and control groups is due only to chance., TRG – Target, REF - Reference

The MICs of fluconazole were significantly decreased when fluconazole was combined with Gla [17]. In the present study, the possible antifungal mechanism was investigated by studying apoptosis in *C. albicans* through a caspase-independent manner. Apoptosis is a very important cellular process that causes cell death in multi-cellular organs and is vital for normal growth and cell retention [18]. Reports of induction of apoptosis in response to Gla therapy in *C. albicans* have been published [8, 19]. Another study showed that farnesol improves apoptosis in *C. albicans* through the activation of caspase, which plays an important physiological role for farnesol in the fungal life cycle and has important outcomes for adaptation and survival [18]. 

Also, typical antifungal agents such as amphotericin B cause apoptosis symptoms in *Rhizopus arrhizus* [20]. Based on our recent data, two genes involved in apoptosis of *Candida* (*MCA1* and *NUC1*) are over-expressed in *C. albicans* cells treated with glabridin, which indicates that apoptosis signals become activated during Gla exposure [13]. Also, overexpression of *MCA1* and *NUC1* genes in *C. glabrata* cells exposed to Gla was observed. Moreover, DNA damage and chromatin density, which indicate the involvement of apoptosis signals, were reported [12]. In the present study, the caspase-independent gene, *AIF*, was over-expressed in response to treatment with Gla in *C. albicans*. Therefore, it is likely that Gla may induce apoptosis through the caspase-independent manner.

## Conclusion

Gla can be used as an antifungal agent against C*andida* isolates. Studying the Gla mechanism of action is recommended by evaluating more genes. Moreover, other assays would be applied in future studies.
